# Supplementary material for: Growth Hormone Receptor Antagonist Markedly Improves Gemcitabine Response in a Mouse Xenograft Model of Human Pancreatic Cancer
Source: Int J Mol Sci. 2024 Jul 6;25(13):7438. doi: 10.3390/ijms25137438 (PMC11242728; doi:10.3390/ijms25137438)
Supplement: Supplementary file 1 [file ijms-25-07438-s001.zip › ijms-3029461-supplementary.pdf]

Supplementary Figures
(in order of narration)

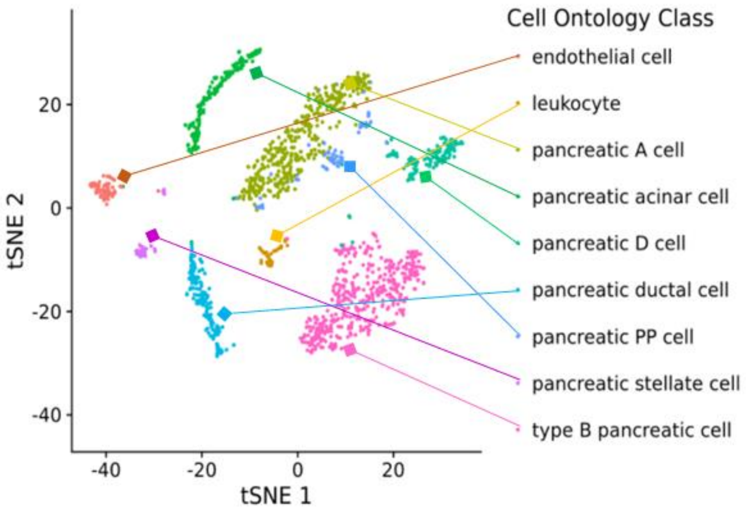

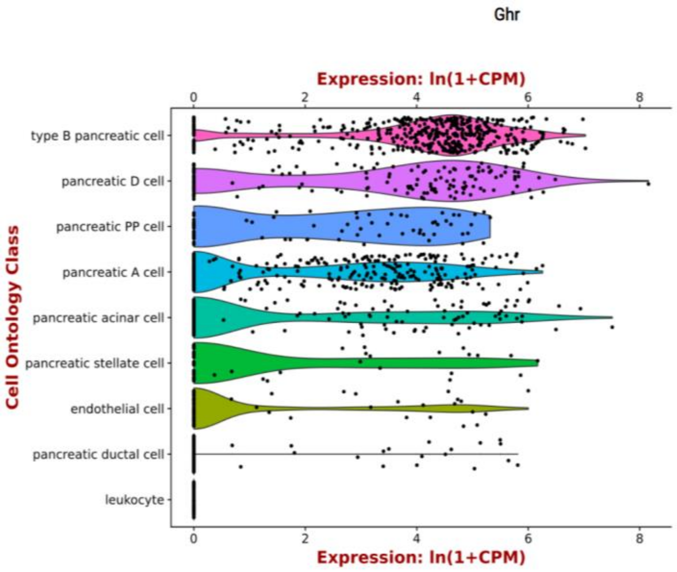

CELL TYPE	MEAN	MEDIAN	# cells	exp% 0	exp% >0
pancreatic B cell	4.0	4.4	449.0	7.8	92.2
pancreatic D cell	3.3	4.0	140.0	22.9	77.1
pancreatic PP cell	2.3	2.7	73.0	32.9	67.1
pancreatic A cell	1.9	1.5	390.0	43.1	56.9
pancreatic ainar cell	1.8	0.0	182.0	56.0	44.0
pancreatic stellate cell	1.4	0.0	49.0	59.2	40.8
endothelial cell	1.2	0.0	66.0	68.2	31.8
pancreatic ductal cell	0.5	0.0	161.0	88.2	11.8
leukocyte	0.0	0.0	54.0	100.0	0.0

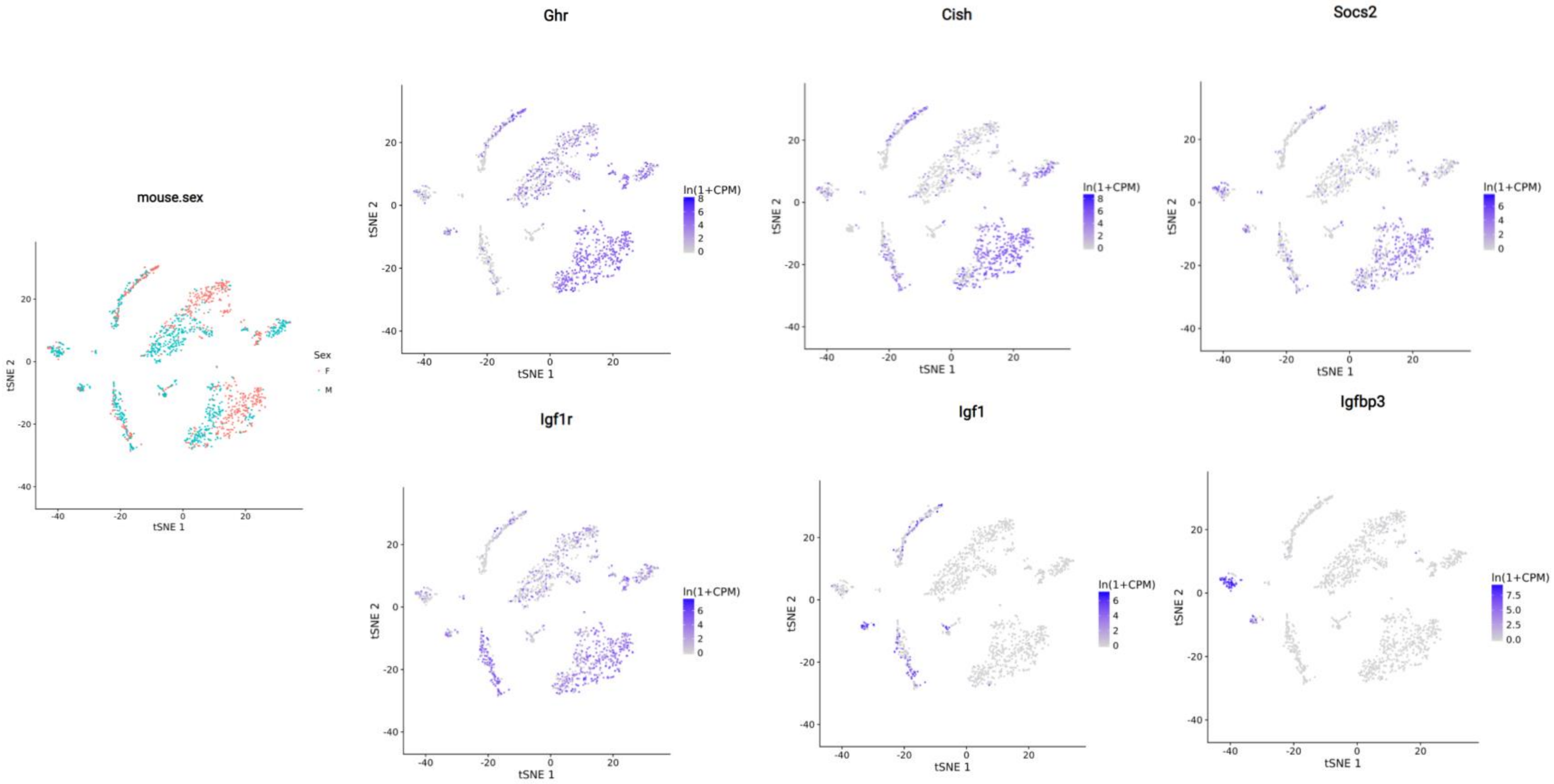

Fig. S1: GHR expression in mouse pancreatic cell types: *Top-left*: Pancreatic cell types in the mouse pancreas as identified by single-cell sequencing using Tabula muris – a compendium of mouse single-cell transcriptomic data from 100,000 cells from 20 organs and tissues of 8 mice. *Top-right*: Violin-plot of GHR RNA expression in the different cell types in the mouse pancreas using Tabula muris. *Bottom*: Ghr, Cish, Socs2, Igf1r, Igf1, and Igfbp3 RNA expression in different cell types in the mouse pancreas using Tabula muris. [Data analyzed from: *The Tabula Muris Consortium*., Schaum, N. *et al. Nature,* 2018. Doi: 10.1038/s41586-018-0590-4]

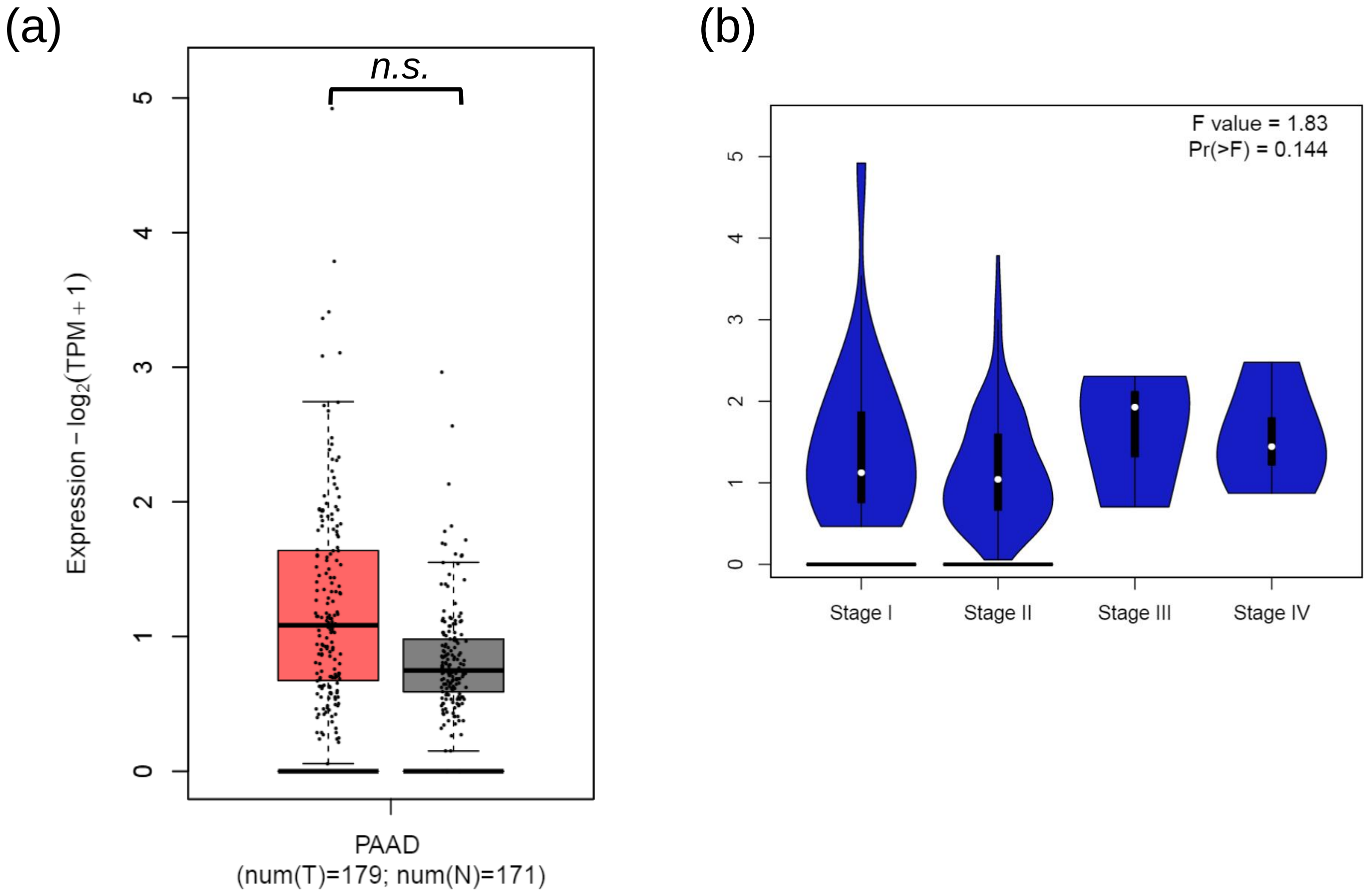

Fig. S2: GHR expression in human pancreatic tumors: (a) GHR RNA expression in PAAD tumor (*red*, n=179, TCGA) vs normal pancreatic tissue (*grey*, n = 171, TCGA + GTEx) in humans. Data generated using GePIA2. Each dot represent one sample. (b) GHR RNA expression across different stages of PDAC in human patients (TCGA) [generated using GePIA2: *Tang et al., Nucleic Acids Research, 2017,* doi: 10.1093/nar/gkx247]

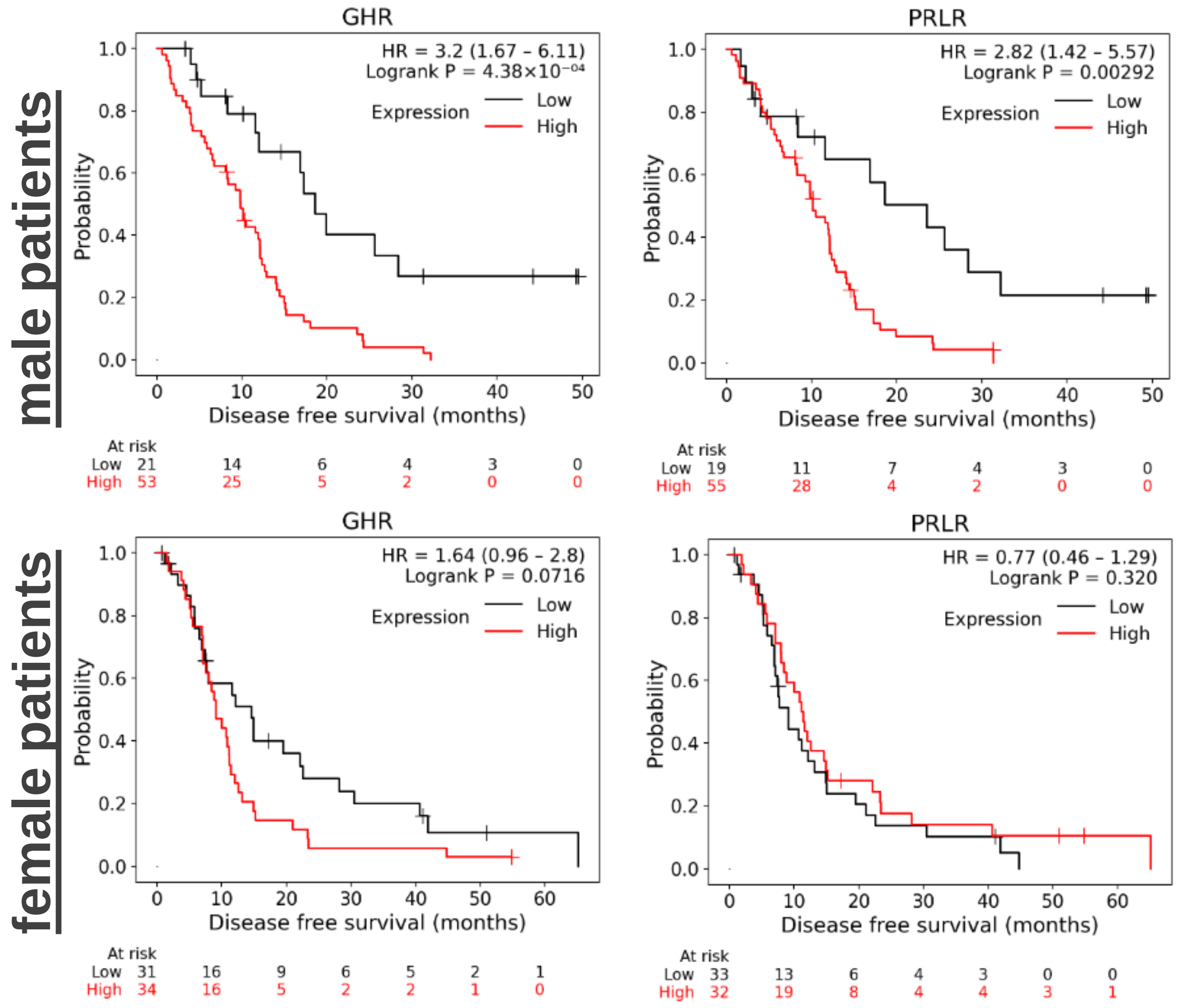

only PDAC	GHR-high	GHR-low	PRLR-high	PRLR-low
patient #:	**107**	**43**	111	39
Hazard ratio (HR):	**2.16**		1.66	
log-rank p:	0.0003		0.0179	
median disease-free survival (months):	**9.1**	**17.3**	9.8	14.8
only PDAC	GHR-high	GHR-low	PRLR-high	PRLR-low
patient #:	53 (male), 34 (female)	21 (male), 31 (female)	55 (male), 32 (female)	19 (male), 33 (female)
Hazard ratio (HR):	3.2 (male), 1.64 (female)		2.8 (male), 0.77 (female)	
log-rank p:	>0.0004(male), 0.07(female)		0.003 (male), 0.320 (female)	
median disease-free survival (months):	9.8 (male), 9.1 (female)	18.6 (male), 14.6 (female)	10.1 (male), 11.1 (female)	23.6 (male), 9.1 (female)

Fig. S3: Correlation of tumoral expression of receptors (GHR and PRLR) of GH with patient **disease-free survival (DFS)** in male and female human pancreatic ductal adenocarcinoma (PDAC) patients. Generated using KMplotter [Data re-analyzed from Posta and Gyorffy, *Clinical and Translational Science,* 2023, doi: 10.1111/cts.13563]

male patients	female patients

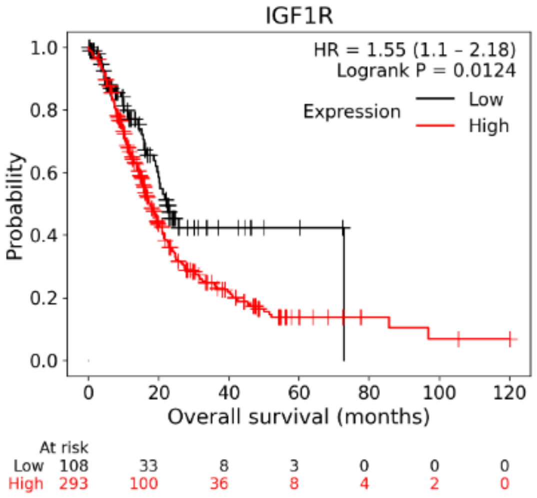

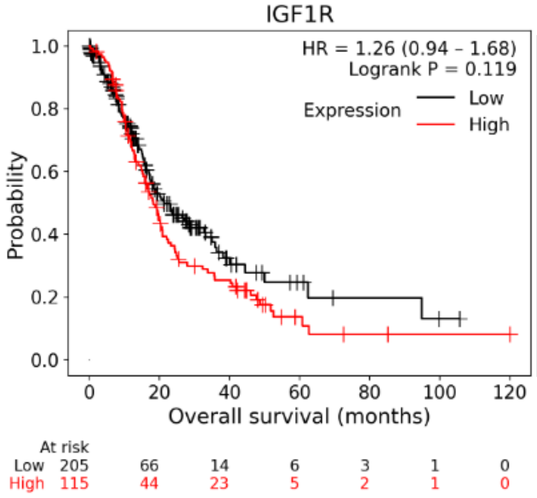

all pancreatic cancer	IGF1R-high	IGF1R-low
patient #:	293 (male), 44 (female)	108 (male), 66 (female)
Hazard ratio (HR):	1.55 (male), 1.26 (female)	
log-rank p:	0.0124 (male), 0.119 (female)	
median overall survival (months):	17.4 (male), 18.2 (female)	22.2 (male), 21.3 (female)

Fig. S4: Correlation of tumoral IGF1R expression with patient median **overall survival (OS)** in patients with pancreatic cancer. Generated using KMplotter [Data re-analyzed from Posta and Gyorffy, *Clinical and Translational Science*, 2023, doi: 10.1111/cts.13563]

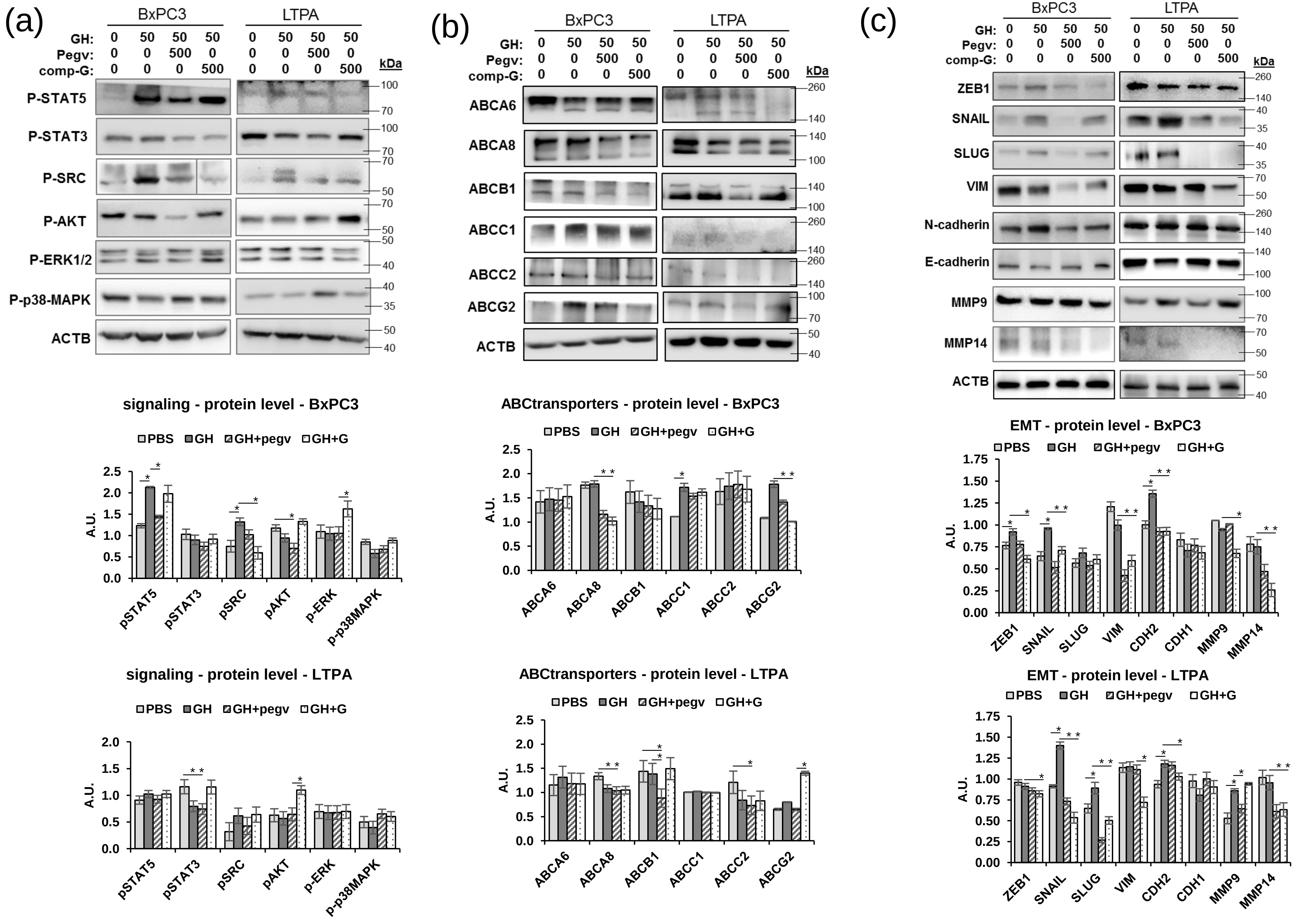

Fig. S5: Western blot of lysates of cultured pancreatic cancer cells treated with GH and GHRAs (Pegv or comp-G) showing (a) activation states of GHR downstream transcription factors, (b) ABC transporters, and (c) EMT factors in BxPC3 and LTPA cell lines (related to Main Fig-1F, 3B, and 4B)

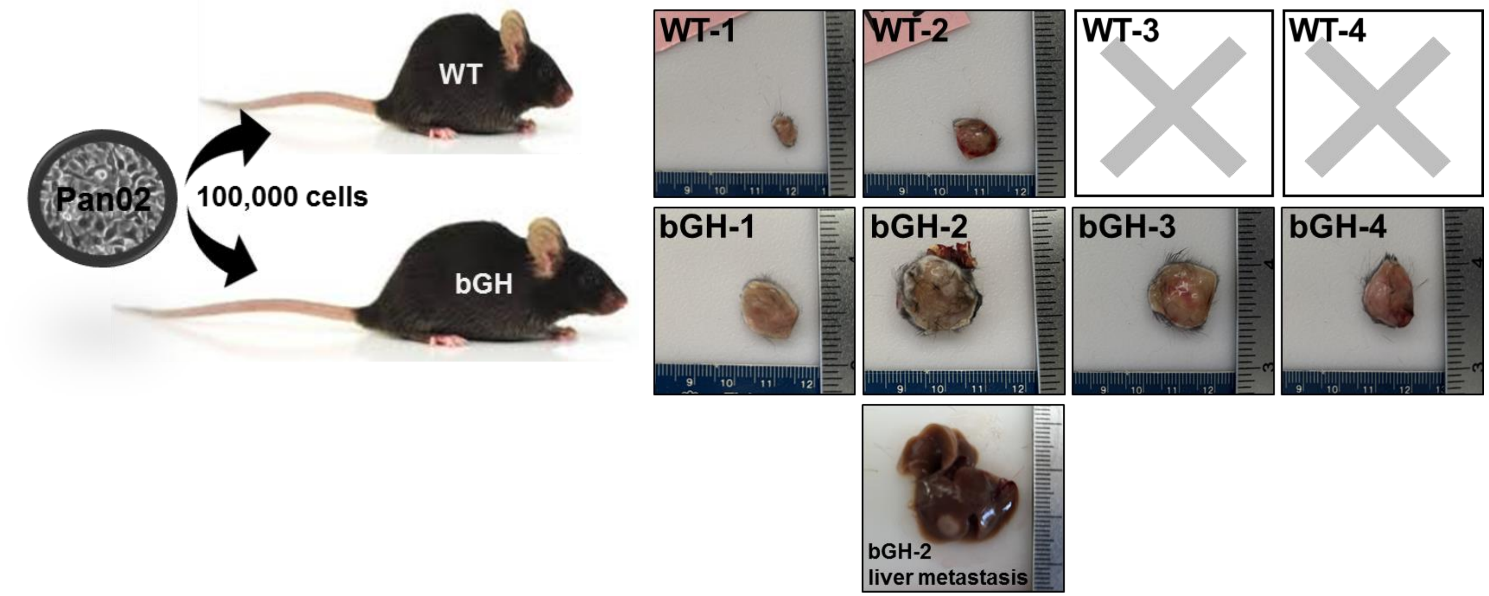

Fig. S6: Pan02 allograft in syngeneic male wild-type of bGH transgenic mice C57BL6 strain: Grafted tumors grew in 4/4 bGH and 2/4 Wt mice. One bGH mouse was also found to have extensive liver tumor (spontaneous or possible metastases).

Fig. S7: Pearson correlation of RNA level of GHR with that of GH/IGF signaling regulators in human PDAC patients (only genes with FDR<0.05 are included, TCGA PDAC dataset).

GH/IGF axis

Query	R^2 value	FDR (BH)
GHR	1.00	1.0E-52
IGF1	0.84	3.3E-38
AKT3	0.74	3.3E-25
FYN / SRC-A member	0.74	5.2E-25
STAT5B	0.74	6.4E-25
SOCS2	0.73	4.6E-24
PTEN	0.69	3.0E-20
IGFBP7	0.68	9.0E-20
JAK1	0.67	8.5E-19
FGR / SRC-A member	0.59	4.2E-14
STAT3	0.59	8.0E-14
IGFBP4	0.58	8.4E-14
IGFBP5	0.57	5.9E-13
HCK / SRC-B member	0.55	2.8E-12
PTPN7	0.55	5.4E-12
SOCS3	0.51	2.6E-10
PTPN5	0.50	4.2E-10
JAK2	0.49	1.3E-09
BLK / SRC-B member	0.49	1.4E-09
PTPN13	0.49	1.6E-09
MAPK1 / ERK2	0.46	2.1E-08
STAT5A	0.44	9.7E-08
PTPN21	0.41	8.1E-07
PTPN22	0.41	8.3E-07
IGF1R	0.39	4.5E-06
CISH	0.38	4.9E-06
PRLR	0.38	5.9E-06
PTPN11	0.36	2.6E-05
PTPN1	0.35	3.9E-05
PTPN9	0.33	1.4E-04
GH1	0.30	5.5E-04

Corr. Coeff
1.0
0.5
0.0
-0.5
-1.0

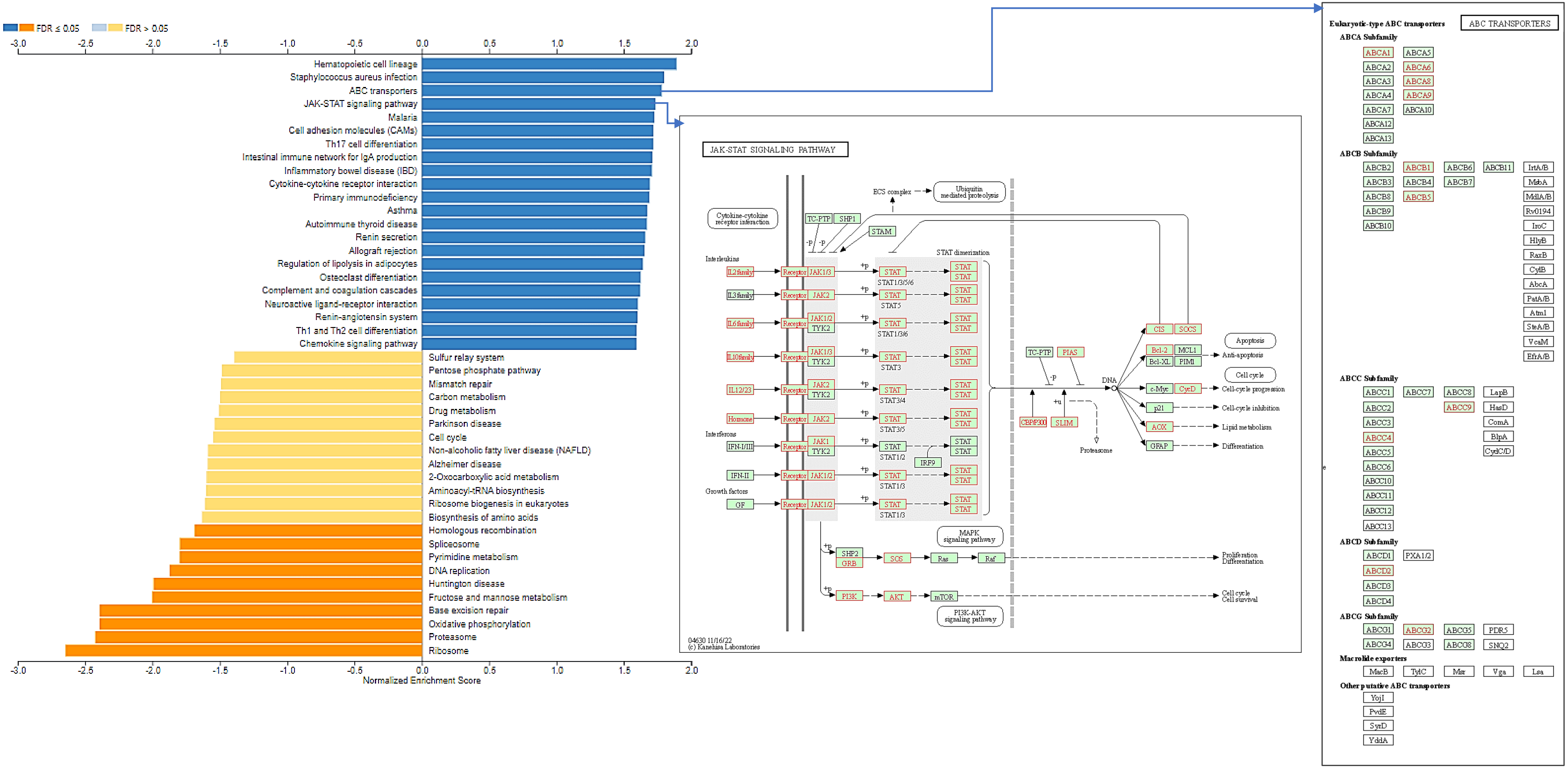

Fig. S8: **KEGG pathways** enriched by genes which are most significantly correlated (positively or negatively) with GHR expression in 154 pancreatic adenocarcinoma patients (TCGA cohort). *Red outline boxes indicate enrichment of genes correlated positively with tumor GHR expression*. [Calculation and Image using Linkedomics]

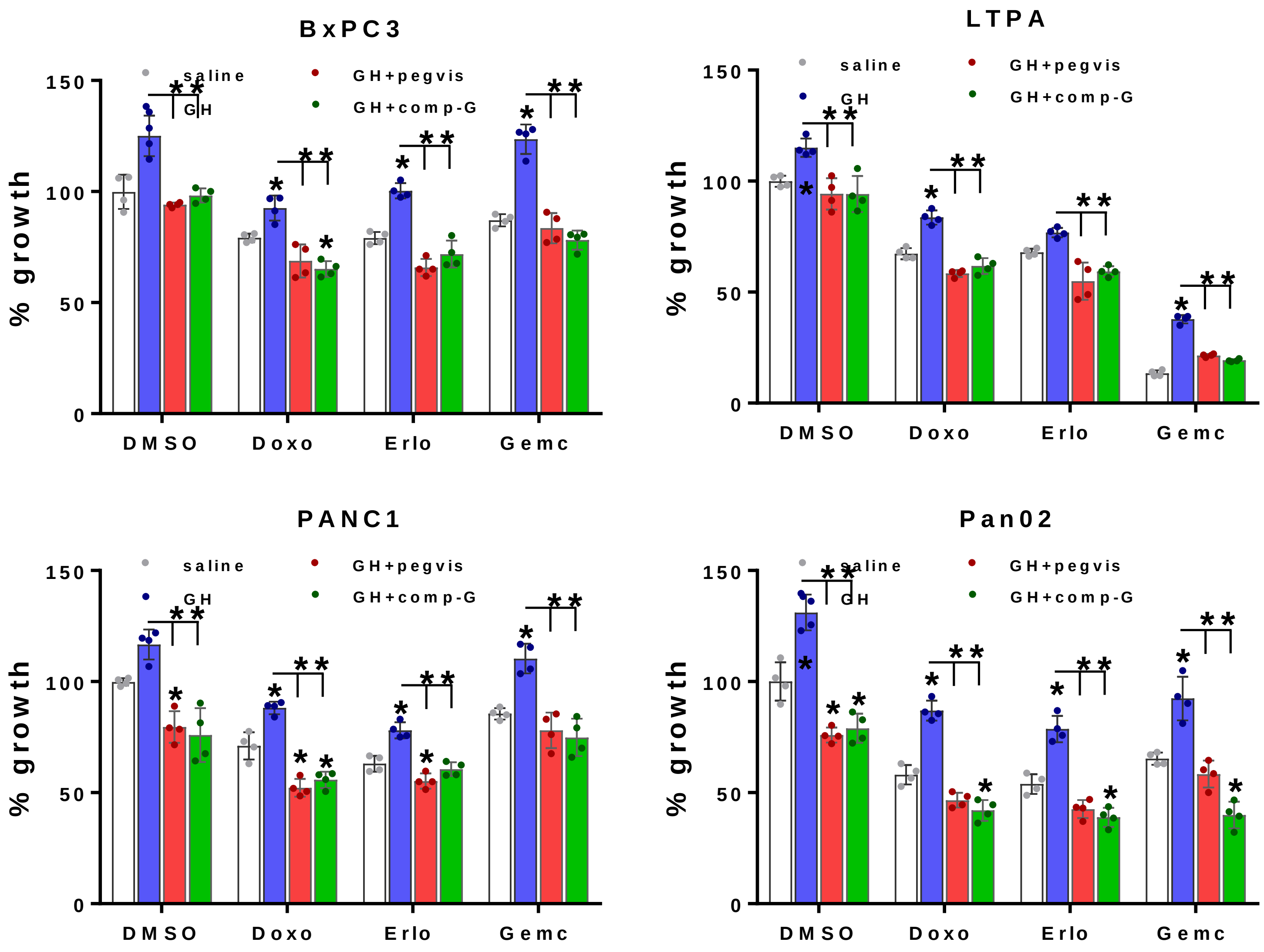

Fig. S9: GHR antagonism improves chemotherapeutic efficacy in pancreatic cancer. (A) Effects of treatments with GH or GH + pegvisomant or GH + compound-G on cytotoxicity of chemotherapies doxorubicin, gemcitabine, erlotinib in human and mouse pancreatic cancel cells in culture.

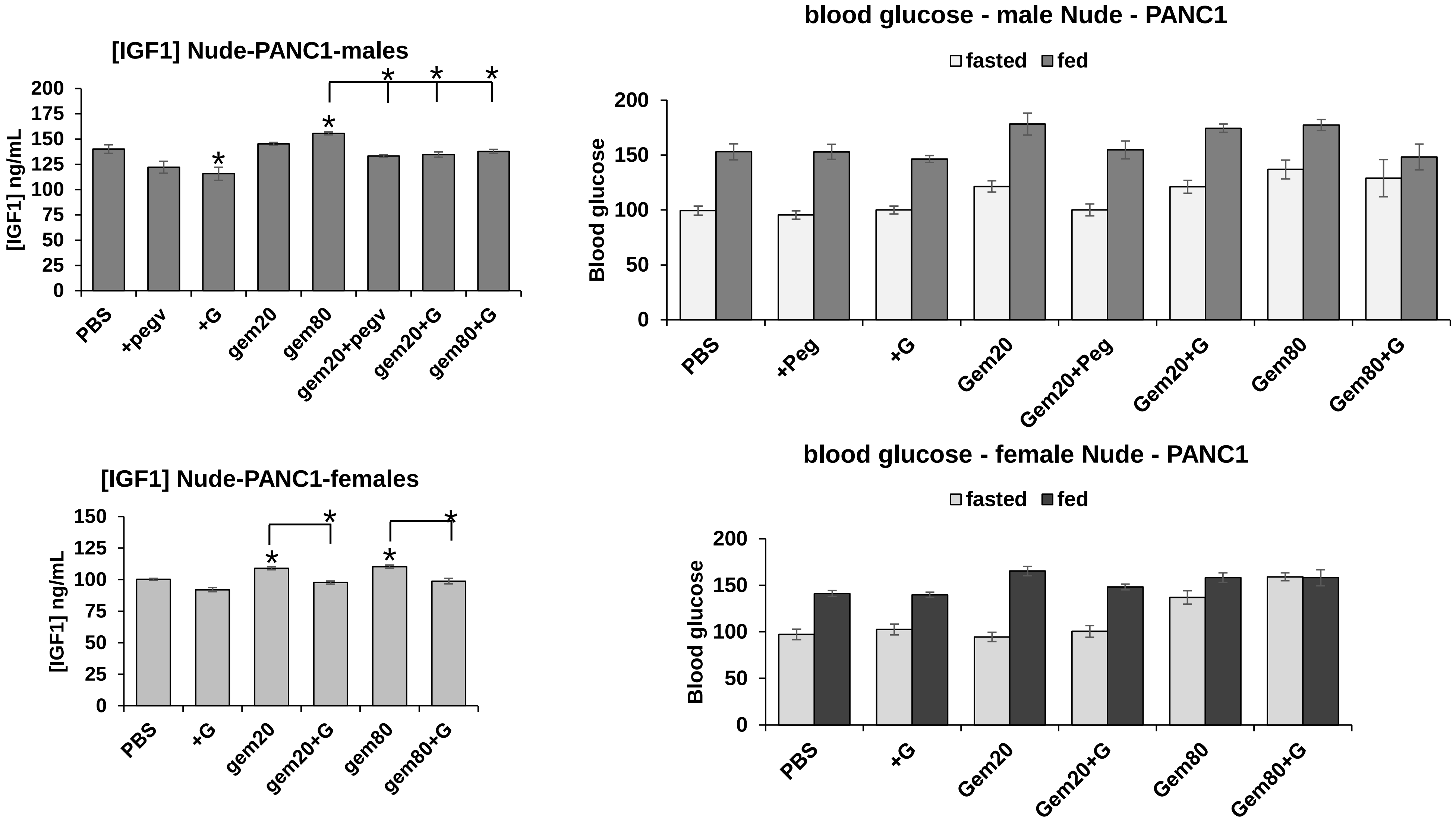

Fig. S10: Serum IGF1 levels, fasted and fed blood glucose levels at end of study of male and female Nude mice with human PANC1 xenografts treated with either gemcitabine (low or high dose), or pegvisomant or compound-G or combinations of gemcitabine and GHRA as mentioned.

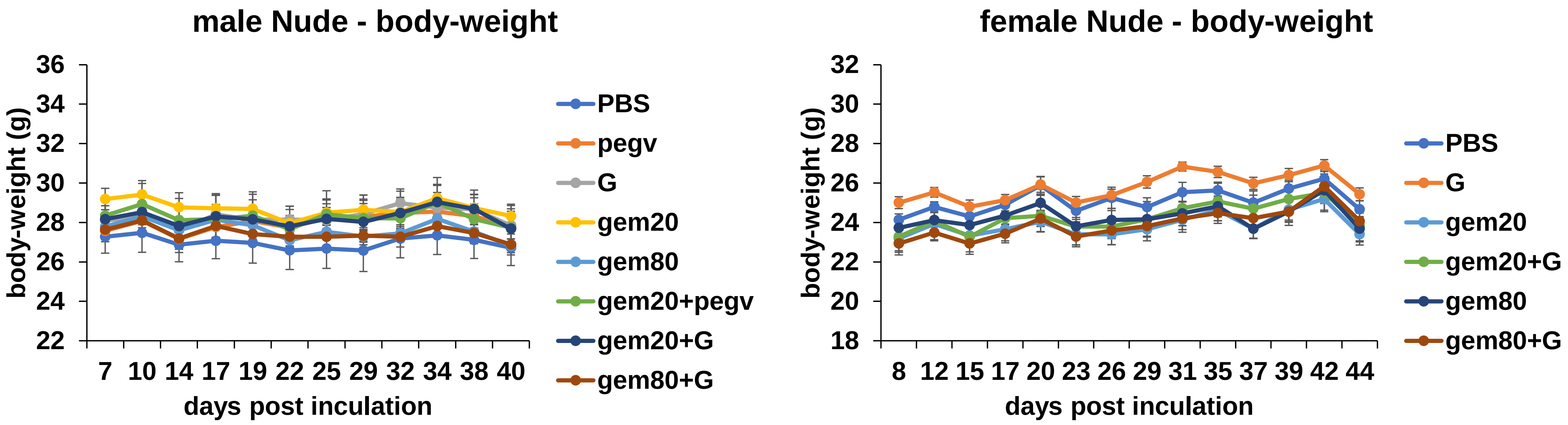

Fig. S11: Body-weight of male (left) and female (right) Nude mice bearing human PANC1 xenografts across treatment period (n=6/group).

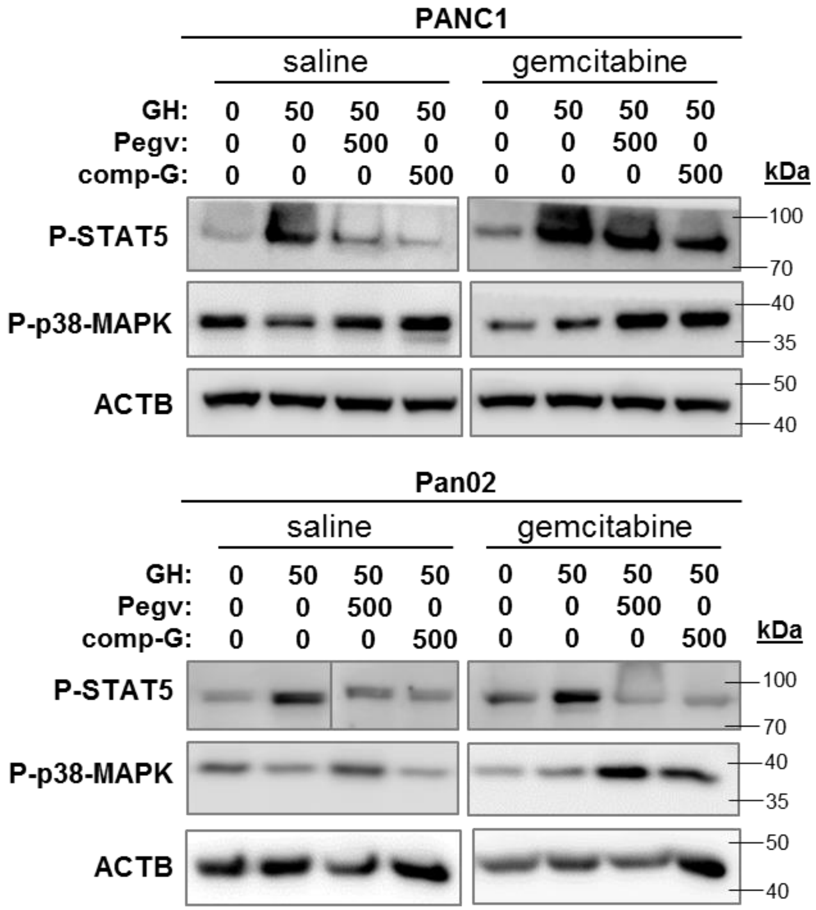

Fig. S12: Western blot of lysates of cultured pancreatic cancer cells treated with GH and GHRAs (Pegv or comp-G) showing activation states of GHR downstream transcription factor STAT5 and that of p38-MAPK which is required for gemcitabine cytotoxic effect.

	Gene (TCGA-PAAD)	Pearson Correl. Coeff. (R^2)	FDR (BH)
import	SLC28A1	**0.103**	0.276854
	SLC28A3	**-0.029**	0.779452
	SLC29A1	**0.283**	0.001121
	SLC29A2	**-0.685**	3.47E-20
activation	DCK	**0.404**	1.39E-06
	CMPK1	**-0.237**	0.007201
	NME4	**-0.181**	0.044934
	RRM2B	**0.402**	1.59E-06
	RRM1	**-0.172**	0.058015
	RRM2	**-0.483**	3.21E-09
deactivation	DCTD	**-0.228**	0.009918
	CDA	**-0.289**	0.00083
	NT5C	**-0.643**	3.08E-17

Corr. Coeff
1.0
0.5
0.0
-0.5
-1.0

Fig. S13: Pearson correlation of RNA level of GHR with that of modulators of gemcitabine action in human PDAC patients (false discovery rate – FDR, was calculated by Benjamini Hochberg (BH) correction from TCGA PDAC dataset).

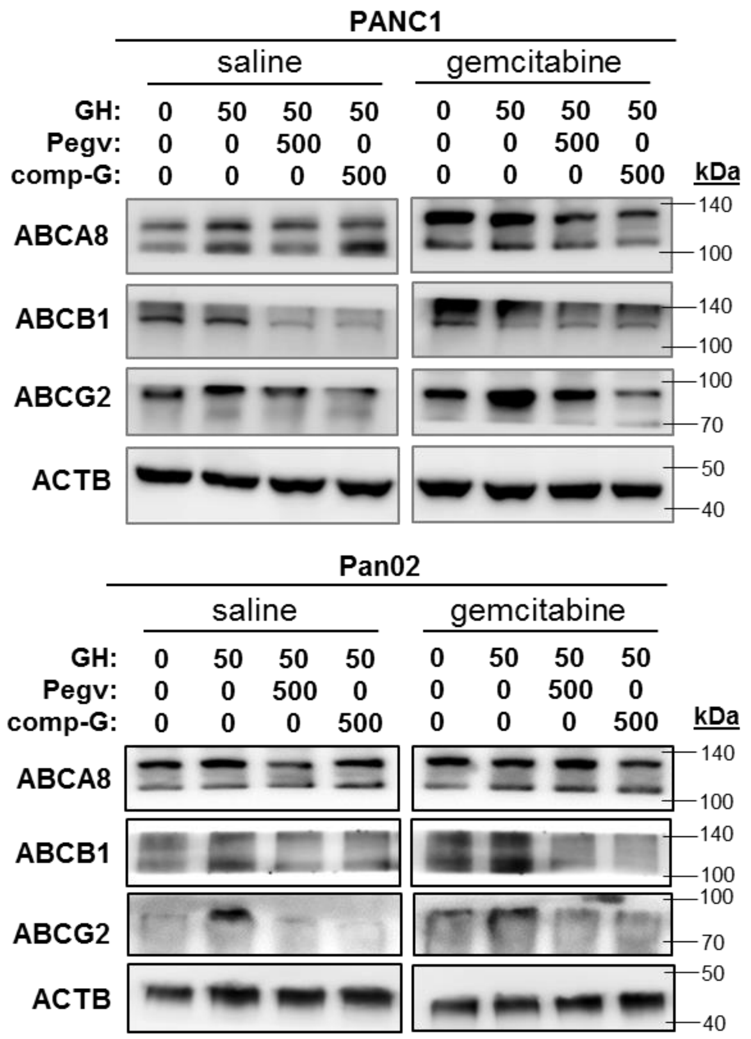

Fig. S14: Western blot of lysates of cultured pancreatic cancer cells treated with GH and GHRAs (Pegv or comp-G) showing expression of ABC-type multidrug transporters.

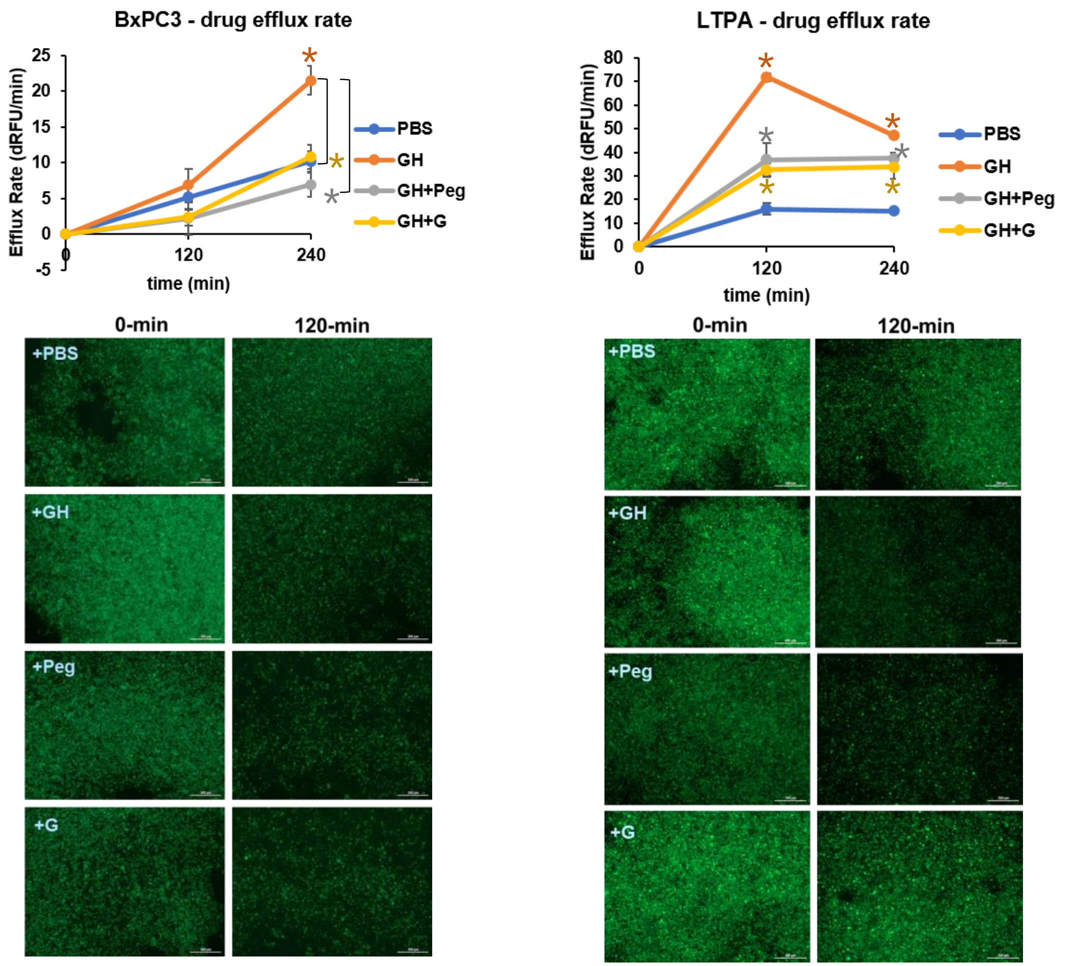

Fig. S15: Drug efflux assay of human and mouse pancreatic cancer cells in culture following treatments with either GH or GH+ GHRAs (pegvisomant or compound-G). Fluorescent green DiOC2 acts as surrogate for drug and is a substrate of ABC-transporters.

C57Bl6-Pan02

Gene	male: bGH vs. Wt
Gh1	1.3
Ghr	1.8
Igf1	4.9
Igf1r	5.3
Prlr	5.4

Nude-PANC1 xenograft

Gene	male: pegv vs. control	male: G vs. control	female: G vs. control
GH1	3.5	2.5	1.0
GHR	0.9	1.1	1.0
Igf1r	0.9	0.9	0.6
IGF1R	1.6	1.1	1.0
PRLR	6.9	6.6	0.9

Nude-PANC1 xenograft

Gene	male: gem20 vs. control	female: gem20 vs. control
GH1	1.4	3.4
GHR	1.7	3.9
Igf1r	1.6	5.2
IGF1R	1.6	5.6
PRLR	3.0	3.5

Nude-PANC1 xenograft

Gene	male: gem20+ pegv vs. gem20	male: gem20+G vs. gem20	female: gem20+G vs. gem20
GH1	1.7	0.9	0.2
GHR	1.1	0.3	0.2
Igf1r	0.8	1.1	0.3
IGF1R	0.7	0.8	0.1
PRLR	1.1	0.4	0.1

Nude-PANC1 xenograft

Gene	male: gem80 vs. control	female: gem80 vs. control
GH1	1.9	0.4
GHR	1.3	0.2
Igf1r	1.8	0.7
IGF1R	1.2	0.5
PRLR	3.1	0.3

Nude-PANC1 xenograft

Gene	male: gem80+G vs. gem80	female: gem80+G vs. gem80
GH1	0.5	1.1
GHR	0.4	0.9
Igf1r	1.0	1.5
IGF1R	0.6	3.2
PRLR	0.3	0.9

fold change
2.0
1.5
1.0
0.8
0.5

Fig. S16: Reverse transcription and real-time quantitative PCR of RNA from xenograft tumors for relative RNA levels and Fold-changes in xenograft tumors in bGH vs WT, and male and female Nude mice with PANC1 tumors and treated with gemcitabine or GHRAs alone or in combination.

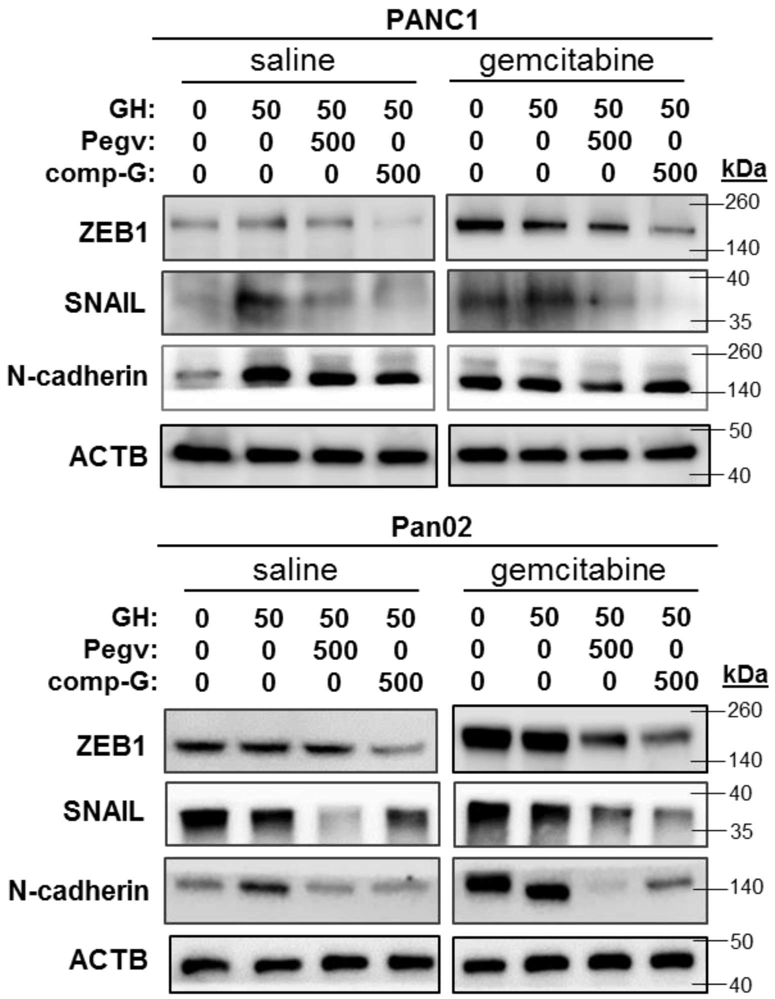

Fig. S17: Western blot of lysates of cultured pancreatic cancer cells treated with GH and GHRAs (Pegv or comp-G) showing expression of epithelial-to-mesenchymal transition (EMT) transcription factors (Zeb1, Snail) and markers (N-cadherin).

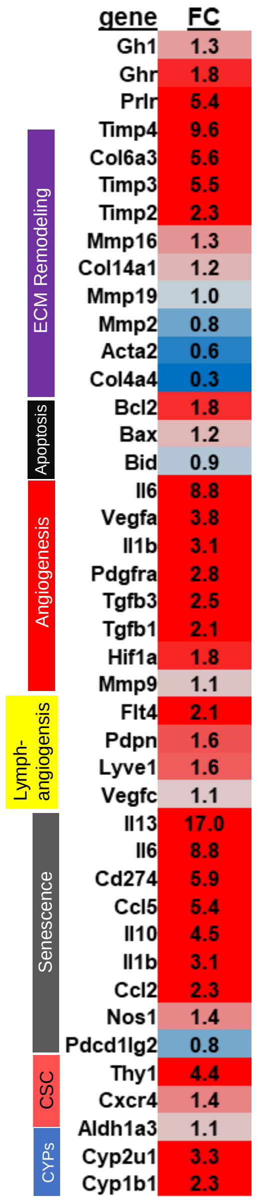

Fig. S18: Heatmap showing fold change (FC) in specific modules of genes affected by tumor-specific actions of GH in xenograft tumors of bGh vs. Wt mice (related to Fig 5H)

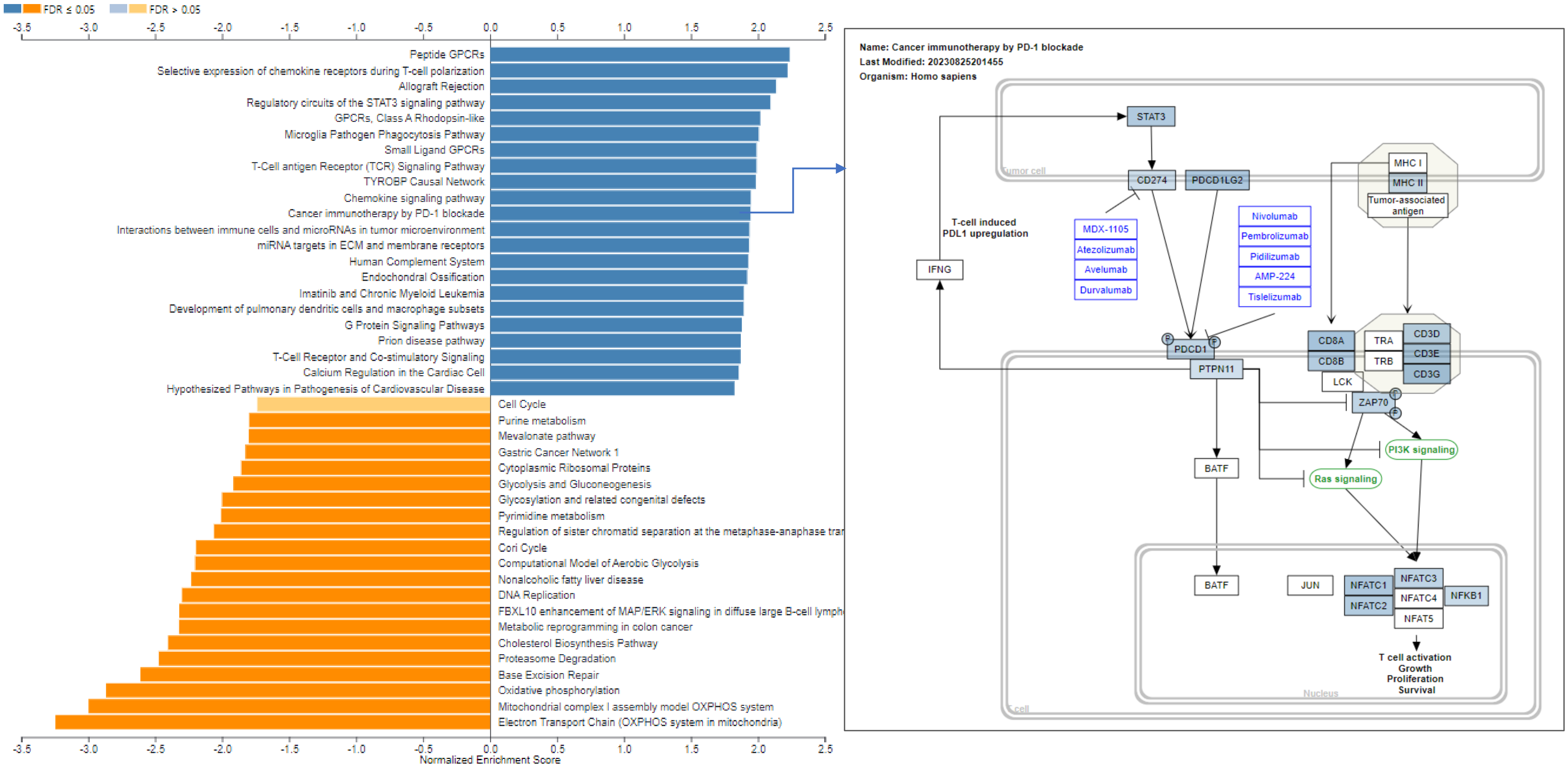

Fig. S19: **Wikipathways** enriched by genes which are most significantly correlated (positively or negatively) with GHR expression in 154 pancreatic adenocarcinoma patients (TCGA cohort). *Blue fill boxes indicate enrichment of genes correlated positively with tumor GHR expression. Blue outline only boxes indicate available immunotherapies to target this pathway*. [Calculation and Image using Linkedomics]

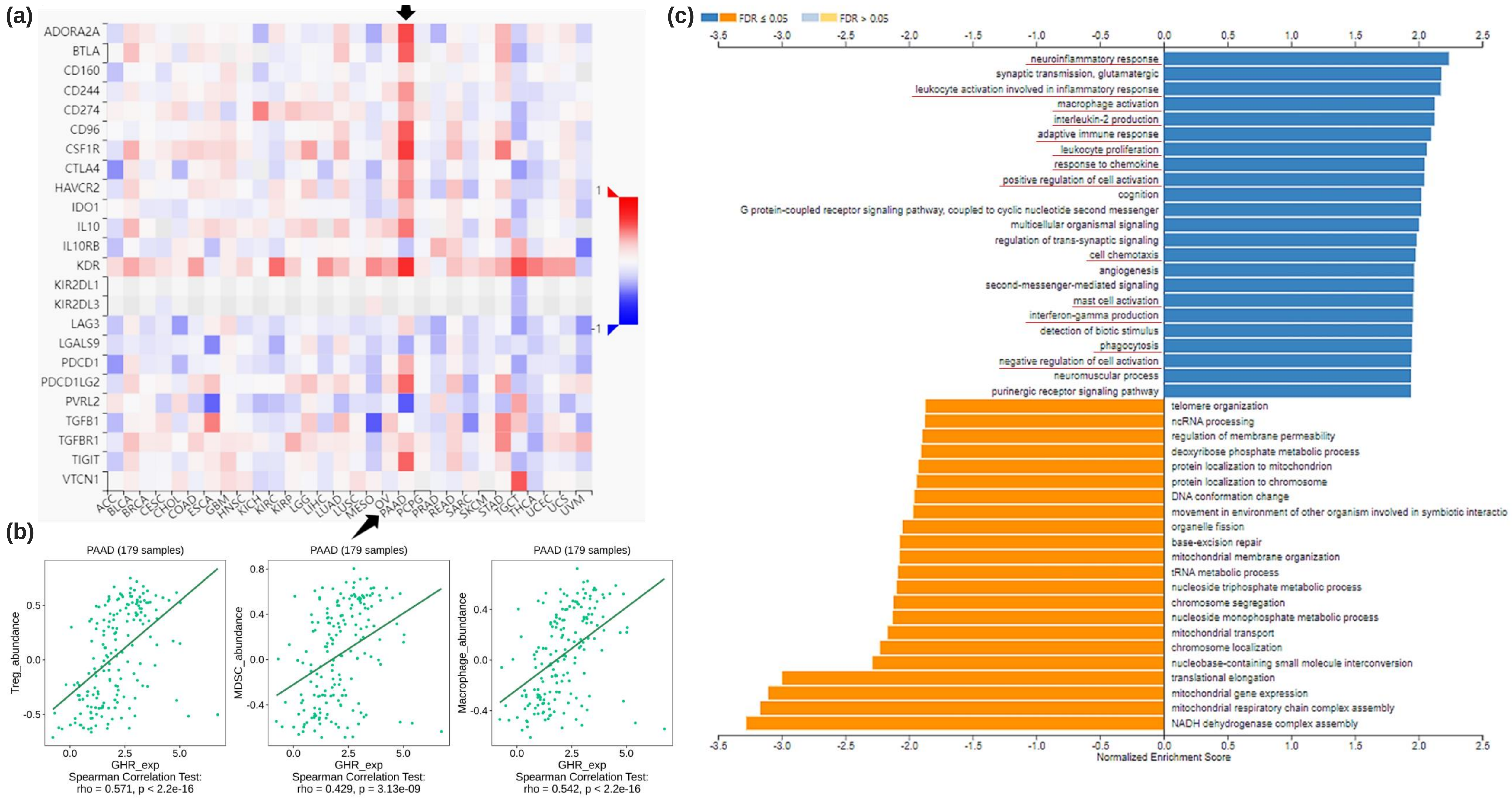

Fig. S20: Bioinformatic analyses of tumor RNA expression and related pathways from patient samples (PDAC, TCGA cohort): **(a)** Immunoinhibitory cytokines in tumors of patients with 30 different cancer types (TCGA cohort; generated using TISIDB platform). **(b)** Spearman correlation of GHR and tumor infiltrating lymphocytes assessed from TIL-specific markers from tumor transcriptome of 179 human patients with PDAC (TCGA cohort; generated using TISIDB platform). **(c)** GSEA (*Gene Ontology: Biological Process*) with genes which correlate positively or negatively with GHR expression in 154 samples from patients with PDAC (TCGA cohort; generated using Linkedomics platform; Red underline indicates tumoral enrichment of immune related pathways by genes correlated positively with tumor GHR expression).

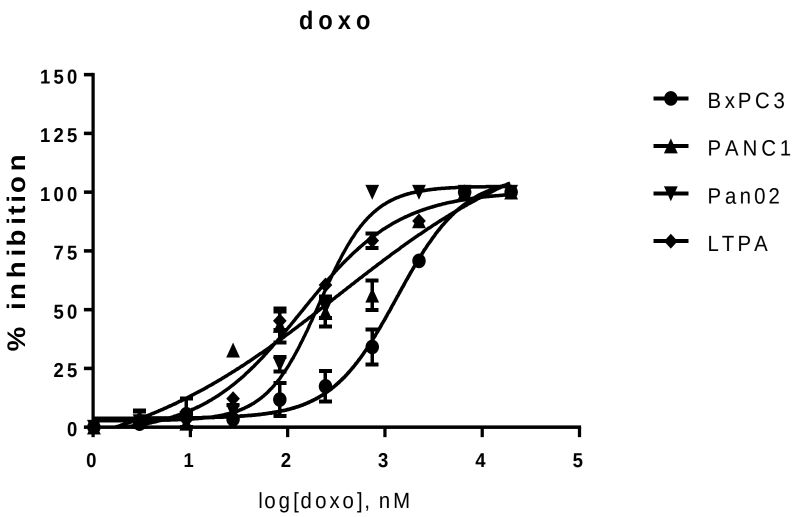

	BxPC3	PANC1	Pan02	LTPA
IC50	1333	330.3	212.3	136.7

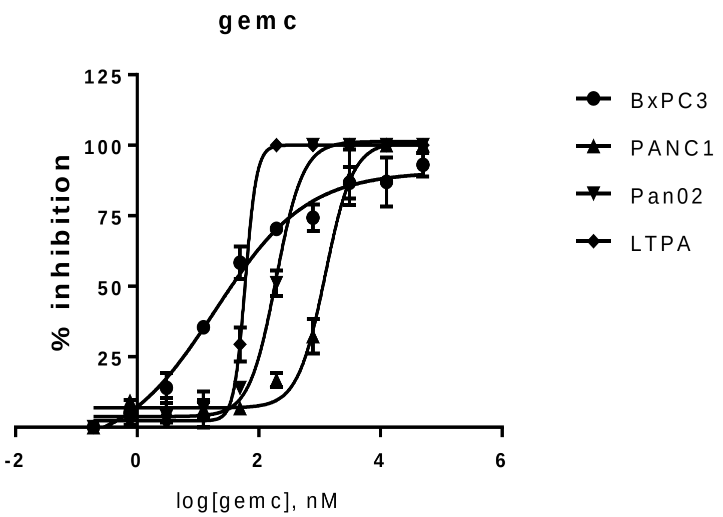

	BxPC3	PANC1	Pan02	LTPA
IC50	19.12	1237	193	60

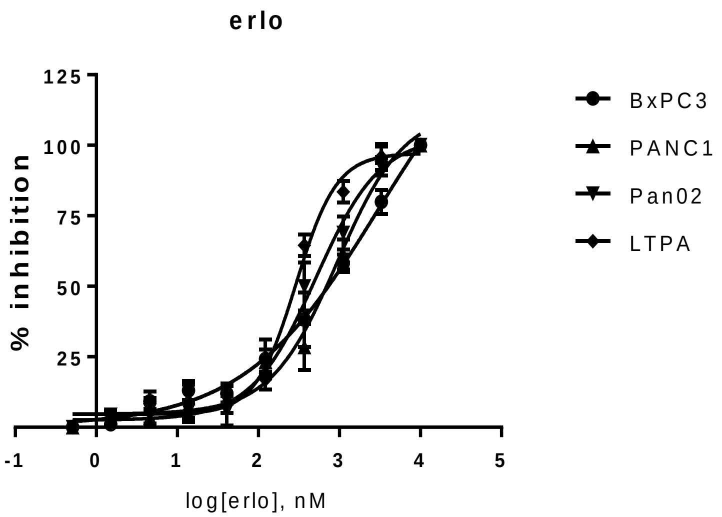

	BxPC3	PANC1	Pan02	LTPA
IC50	3047	937.4	512.1	292.1

Fig. S21: EC50s (in nM) of the chemotherapies (doxorubicin, gemcitabine, erlotinib) used in the study in all four human and mouse pancreatic cancer cell lines. Resazurin-resorufin cell viability assay (48-hr) was employed for 10-dose EC50 determination in 96-well plate format with 5000 cells/well. Percent inhibition was calculated and nonlinear regression (variable slope – four parameters) was used to calculate EC50 in Graphpad Prizm.

target gene	forward sequence (5' --> 3')	reverse sequence (5' --> 3')
**Abca1**	GGTCTCCAGAAGGTATTTTTG	TCAGGATGTCCATGTTGTAG
**Abca6**	CTGGGATTATAGAAGTCACAAC	CGAGGAGAAATAAAGCAAGC
**Abca8a**	GGCTTATCTCTGATCTCTTTG	GACTGAGCAAACTTAGAAGC
**Abca9**	CTTTCCTCATGAGTGTGTTG	GTCCAAATGTATAAGCTGGG
**Abcb1a**	AGGACAAAAGAAGGAACTTG	GATAAGGAGAAAAGCTGCAC
**Abcc1**	GTCTATCGTAAGGCTCTTTTG	GACCAGATCATGTTAATGTACG
**Abcc2**	CGTATATAAGAAGGCACTAACC	CAATCTGTAAAACACTGGACC
**Abcc4**	AAACAAAGTCATCCTGTTCG	CAGAAAGTTCTTGATCCTCC
**Abcc5**	TAGGGGAGCTCATCAATATC	ATAAATCATGCCCAAGATGG
**Abcc9**	TGAAGAGCAGAAGAAAAAGG	TTTTCATTTACTCGCTGGAC
**Abcg2**	AAGAGCCAGTCTATGTTACC	AAACTCCAGCTCTATTTTGC
**Cdh1**	CATGTTCACTGTCAATAGGG	GTGTATGTAGGGTAACTCTCTC
**Cdh2**	GAGTTTACTGCCATGACTTTC	TCCACCACTGATTCTGTATG
**Cldn1**	TTTTAATTTCAGGTCTGGCG	CAAATTCATACCTGGCATTG
**Fgfbp1**	ACTCACAGAAAGGTGTCC	CTGCTTCTCTGCTTATTCTG
**Snai1**	AGTTGACTACCGACCTTG	AAGGTGAACTCCACACAC
**Snai2**	GACACATTAGAACTCACACTG	GACATTCTGGAGAAGGTTTTG
**Tgfb1**	GGATACCAACTATTGCTTCAG	TGTCCAGGCTCCAAATATAG
**Tgfb3**	CTCAGTGGAGAAAAATGGAAC	GGTCGAAGTATCTGGAAGAG
**Twist1**	GAGACCTAGATGTCATTGTTTC	GAATTTGGTCTCTGCTCTTC
**Twist2**	CGCATACTCCTGTTCTTTAC	CTCTTTATTGTTCCTGGGTG
**Vim**	GAACCTGAGAGAACTAACC	GATGCTGAGAAGTCTCATTG
**Zeb1**	GAAACCAGGATGAAAGACAAG	TTCCGAGTTTTCTTTTTGGG
**Gh1**	TCCAGTCTGTTTTCTAATGC	TCGAACTCTTTGTAGGTGTC
**Ghr**	ACTGTCCAGTGTACTCATTG	CTGGATATCTTCTTCACATGC
**Igf1**	GACAAACAAGAAAACGAAGC	ATTTGGTAGGTGTTTCGATG
**Igf1r**	AGAACCGAATCATCATAACG	TTTTAAATGGTGCCTCCTTG
**Prlr**	CAAAAGTATCTTGTCCAGACTC	AGGTCATCATGCTATAACCC

target gene	forward sequence (5' --> 3')	reverse sequence (5' --> 3')
**ABCA6**	CCATATGCTATGGGAATCATC	AGCTGAGAAATCTTCTTTCC
**ABCA8**	TCATTATGGCCCTTTTCTTG	TTAAGAAAGCCAAAGCTACC
**ABCA9**	CCCAGCTTATACATTTGGAC	ACCAACATGAAAAGAGTAGC
**ABCB1**	CGTTGAAGAGTAGAACATGAAG	TTGCACCTCTCTTTTATCTG
**ABCC1**	AGCAGAAAAATGTGTTAGGG	TACCCACTGGTAATACTTGG
**ABCC2**	AAATTGCTGATCTCCTTTGC	GATAGCTGTCCGTACTTTTAC
**ABCC4**	ATGGAGATAGGAATATCGTGC	TCCTCAGTGATGAGAACAAC
**ABCC5**	CCTGTATGACTTTTTCGTGG	CCACAGTCTCTCTAGTCTTC
**ABCC9**	GAATTTCAGAAACCCTCTCATC	AGAGCCAAGAAGAGAAGAAC
**ABCG2**	AAAGCCACAGAGATCATAGAG	GATCTTCTTCTTCTTCTCACC
**CDH1**	CCGAGAGCTACACGTTC	TCTTCAAAATTCACTCTGCC
**CDH2**	CTGGAACATATGTGATGACC	TGTAAACATGTTGGGTGAAG
**CLDN1**	TTGGCATGAAGTGTATGAAG	ACCTGCAAGAAGAAATATGC
**EPAS1**	GTGAGATTGAGAAGAATGACG	CTTGGTGAATAGGAAGTTACTC
**FGFBP1**	TGATGAGGGAAAAGGAGAAC	AACACTCACTGGGTTTTTAC
**SNAI1**	CTCTAATCCAGAGTTTACCTTC	GACAGAGTCCCAGATGAG
**SNAI2**	CAGTGATTATTTCCCCGTATC	CCCCAAAGATGAGGAGTATC
**TGFB1**	AACCCACAACGAAATCTATG	CTTTTAACTTGAGCCTCAGC
**TGFB3**	TGTTGAGAAGAGAGTCCAAC	ATCACCTCGTGAATGTTTTC
**TWIST1**	CTAGATGTCATTGTTTCCAGAG	CCCTGTTTCTTTGAATTTGG
**TWIST2**	CATAGACTTCCTCTACCAGG	CATCATTCAGAATCTCCTCC
**VIM**	GGAAACTAATCTGGATTCACTC	CATCTCTAGTTTCAACCGTC
**ZEB1**	AAAGATGATGAATGCGAGTC	TCCATTTTCATCATGACCAC
**GH1**	AGGAAACACAACAGAAATCC	TTAGGAGGTCATAGACGTTG
**GHR**	CTCCTCAAGGAAGGAAAATTAG	GTGGAATTCGGGTTTATAGC
**IGF1**	TTATTTCAACAAGCCCACAG	AATGTACTTCCTTCTGGGTC
**IGF1R**	AGGGAATTACTCCTTCTACG	TTTATGTCCCCTTTGCTTTG
**PRLR**	CAAGTCAAGAGAGAGAACAG	GATGTTGTTATCCATGACCC

Fig. S22: List of primer pairs and sequences used for the current study

Target protein	Target species	Host species	Vendor	Catalog Number
GHR	Human	Rabbit	Abcam	Ab65304
GH	Human	Rabbit	Abcam	Ab155974
HSP90	Human, mouse	Rabbit	CST	4874
P-STAT5a/b	Human, mouse	Rabbit	R&D Systems	MAB41901
P-STAT3	Human, mouse	Rabbit	CST	9145
P-SRC	Human, mouse	Rabbit	CST	2101
P-AKT	Human, mouse	Rabbit	CST	9271
P-ERK1/2	Human, mouse	Rabbit	CST	9102
P-p38-MAPK	Human, mouse	Rabbit	CST	4511
β-actin	Human, mouse	Rabbit	CST	4970
ABCA6	Human, mouse	Rabbit	Invitrogen	PA5-104402
ABCA8	Human, mouse	Rabbit	Invitrogen	PA5-36429
ABCB1	Human, mouse	Rabbit	Invitrogen	MA5-32282
ABCC1	Human	Rabbit	CST	72202
ABCC2	Human	Rabbit	CST	12559
ABCG2	Human, mouse	Rabbit	CST	4477
ZEB1	Human, mouse	Rabbit	CST	70512
SNAIL	Human, mouse	Rabbit	CST	3879
SLUG	Human, mouse	Rabbit	CST	9585
VIMENTIN	Human, mouse	Rabbit	CST	5741
N-CADHERIN	Human, mouse	Rabbit	CST	13116
E-CADHERIN	Human, mouse	Rabbit	CST	3195
MMP9	Human	Rabbit	CST	13667
MMP14	Human, mouse	Rabbit	Invitrogen	MA5-32076
Secondary Antibody	Rabbit	Goat	CST	7074

Fig. S23: List of antibodies used for the current study
